# Supplementary material for: Regulation of Fungal Morphogenesis and Pathogenicity of Aspergillus flavus by Hexokinase AfHxk1 through Its Domain Hexokinase_2
Source: J Fungi (Basel). 2023 Nov 4;9(11):1077. doi: 10.3390/jof9111077 (PMC10671980; doi:10.3390/jof9111077)
Supplement: Supplementary file 1 [file jof-09-01077-s001.zip › jof-2660282-supplementary.pdf]

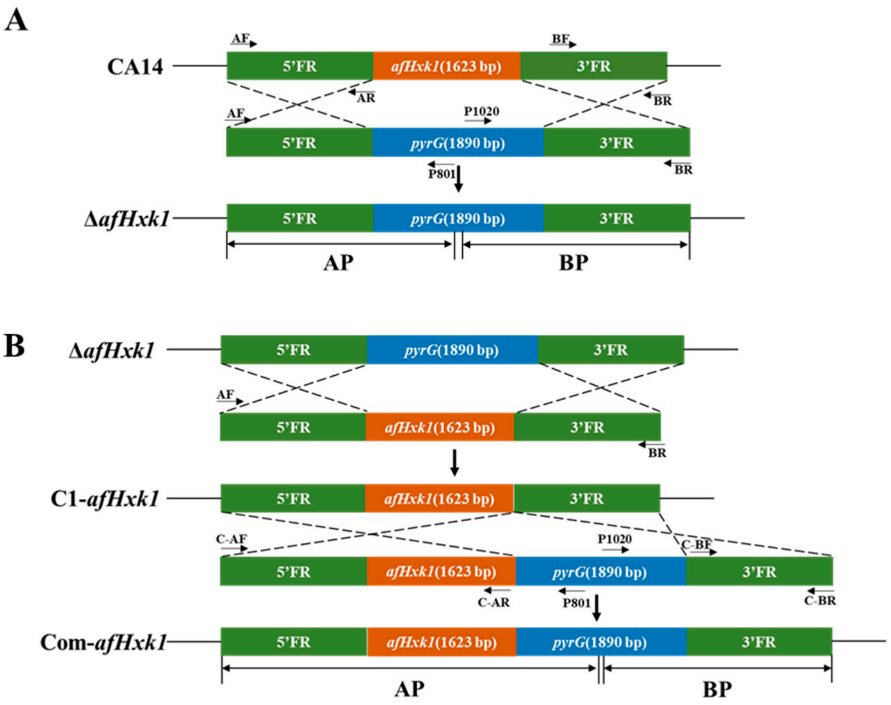

Figure S1. The scheme of fungal strain construction. (A)The principle of constructing the knockout strain Δ*afHxk1*. (B)The construction principle for the complementary strain Com-*afHxk1*.

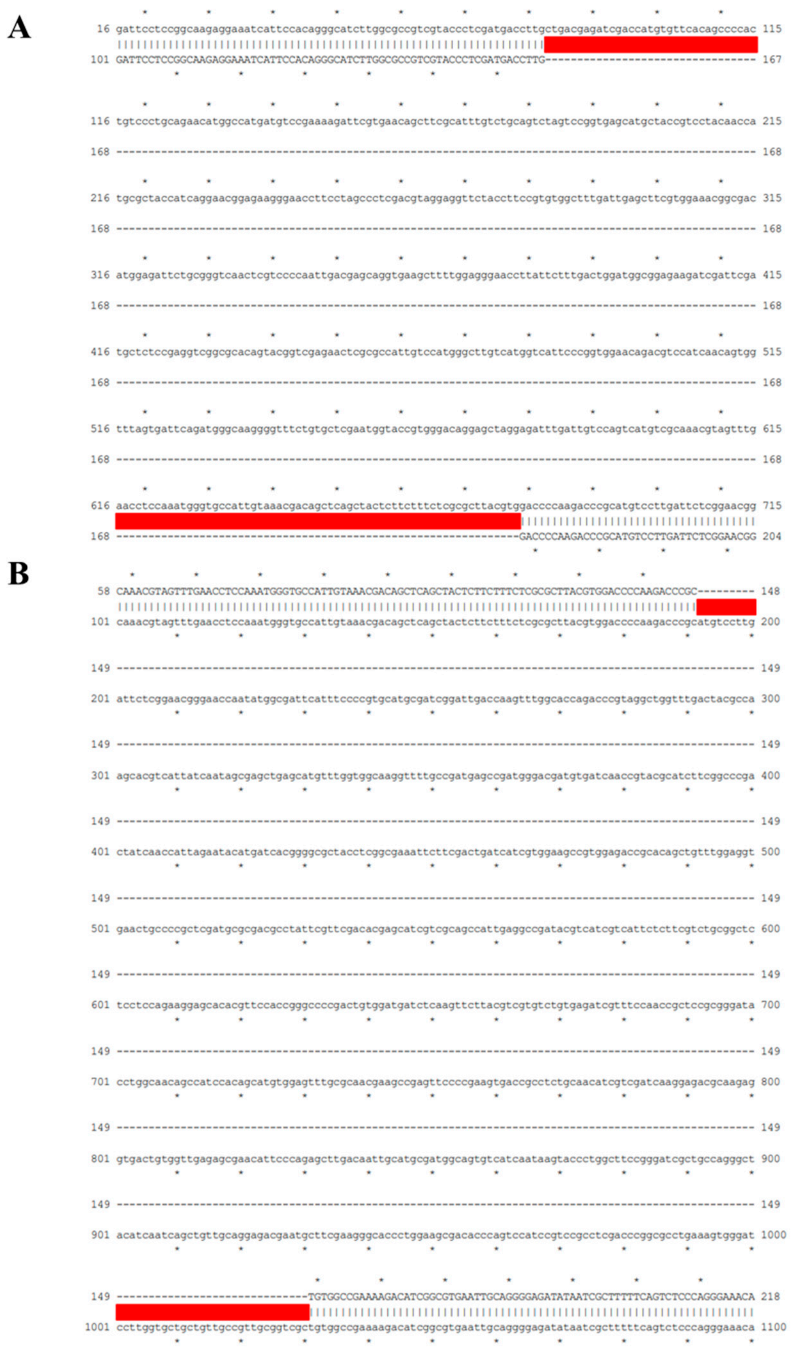

Figure S2. The sequencing results of domain deletion strains. (A) The sequencing result of *afHxk1*$^{\Delta D1}$. (B) The sequencing results of *afHxk1*$^{\Delta D2}$. Sequencing are carried out in Tsingke Biotechnology Co., Ltd..

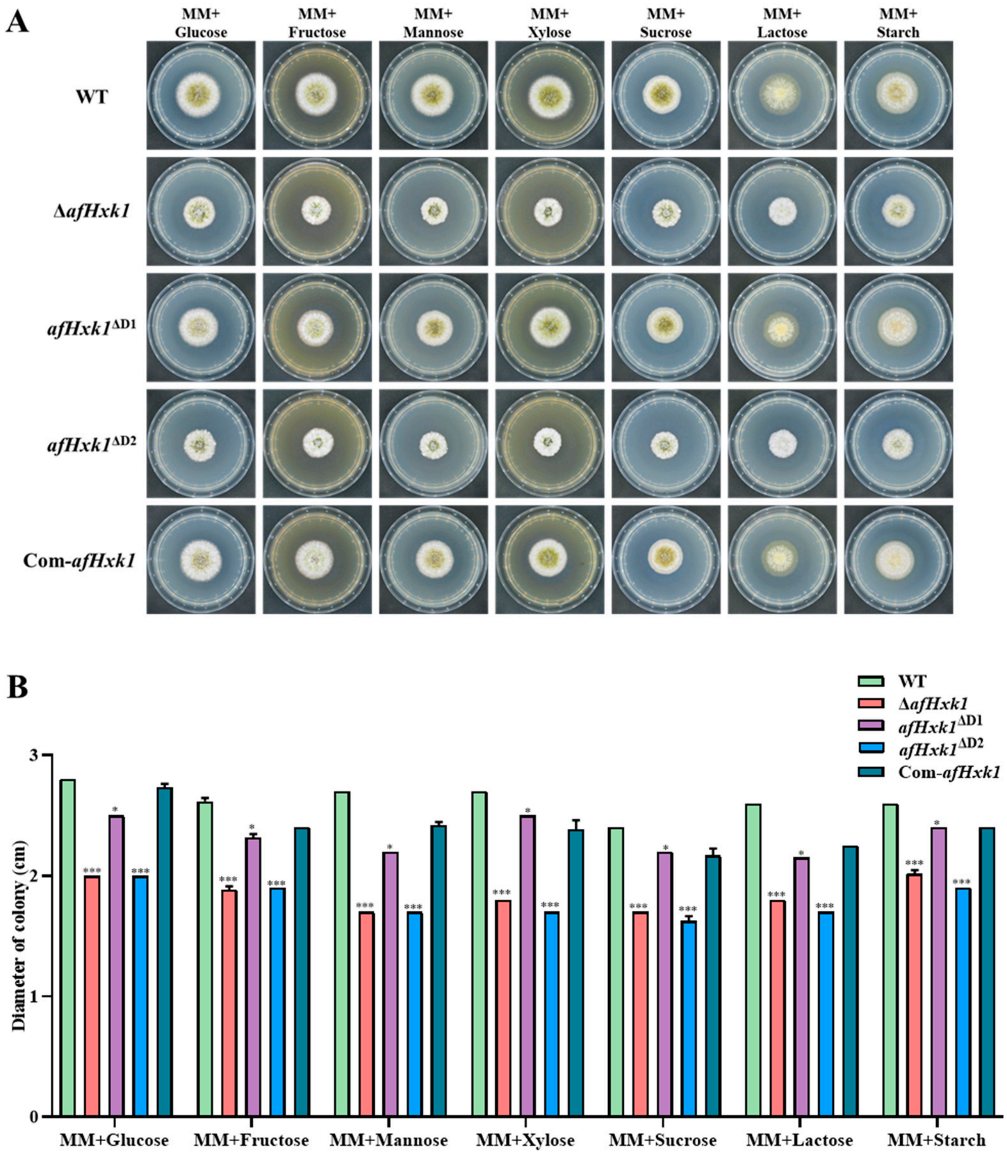

Figure S3. The role of AfHxk1 on utilization capacity of *A. flavus* to different carbon sources. (A) The colonies of fungal strains on MM media with different carbon sources. The fungal strains (WT, Δ*afHxk1* *afHxk1*$^{\Delta D1}$, *afHxk1*$^{\Delta D2}$ and Com-*afHxk1*) were inoculated on MM with 10 g/L glucose, fructose, mannose, xylose, sucrose, lactose, and starch, respectively) for 4 d. (B)Statistics analysis of the diameter on the above media. *, *** means significant difference $P < 0.05$, $P < 0.001$, respectively.

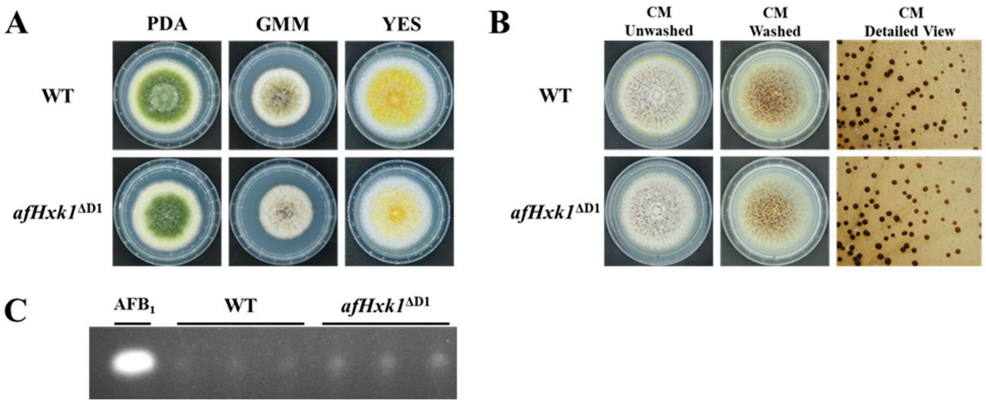

Figure S4. Domain 1 of AfHxk1 doesn't take part in the regulation of fungal morphogenesis and aflatoxin bio-synthesis. (A) The growth of WT and *afHxk1*$^{\Delta D1}$ on the PDA for 4 d. (B) The growth of WT and *afHxk1*$^{\Delta D1}$ on the CM for 7 d. (C) The TLC analysis of AFB1 yield from WT and *afHxk1*$^{\Delta D1}$ inoculated in PDB for 7 d.

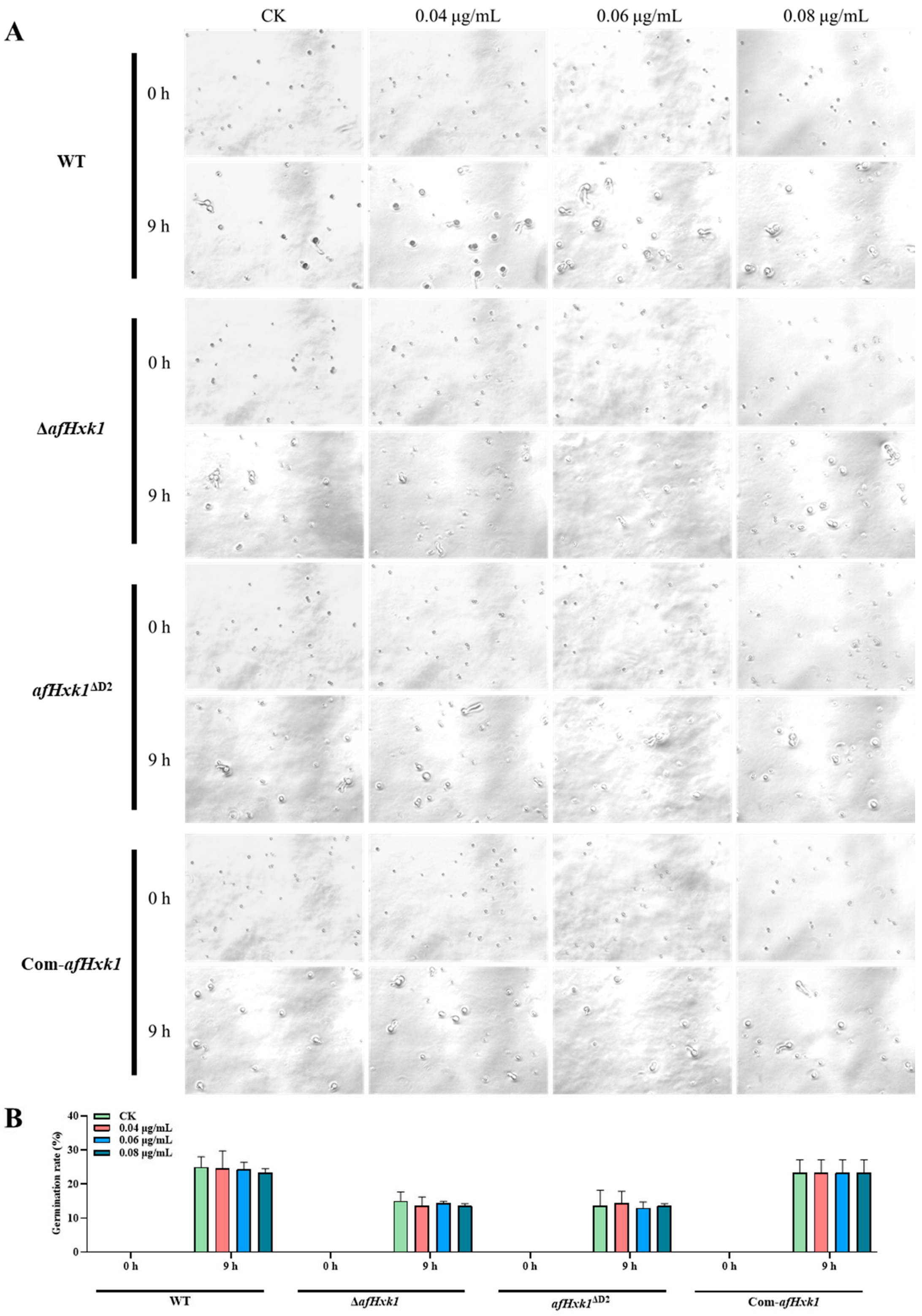

Figure S5. Spore germination under different concentrations of AFB$_1$. (A)The germination of spores under 1/300 (v/v) diluted methanol (Control group, CK) and 0.04 μg/mL AFB$_1$, 0.06 μg/mL AFB$_1$, 0.08 μg/mL AFB$_1$. Each AFB$_1$ concentration contains 1/300 (v/v) diluted methanol. Each strain (WT, Δ*afHxk1*, *afHxk1*$^{\Delta D2}$ and Com-*afHxk1*) was inoculated on PDA media for 0 h and 9 h. Then they

were further observed by microscope. (B) Statistics of germination rate of the above each fungal strain on 0 h and 9 h according to the panel A.

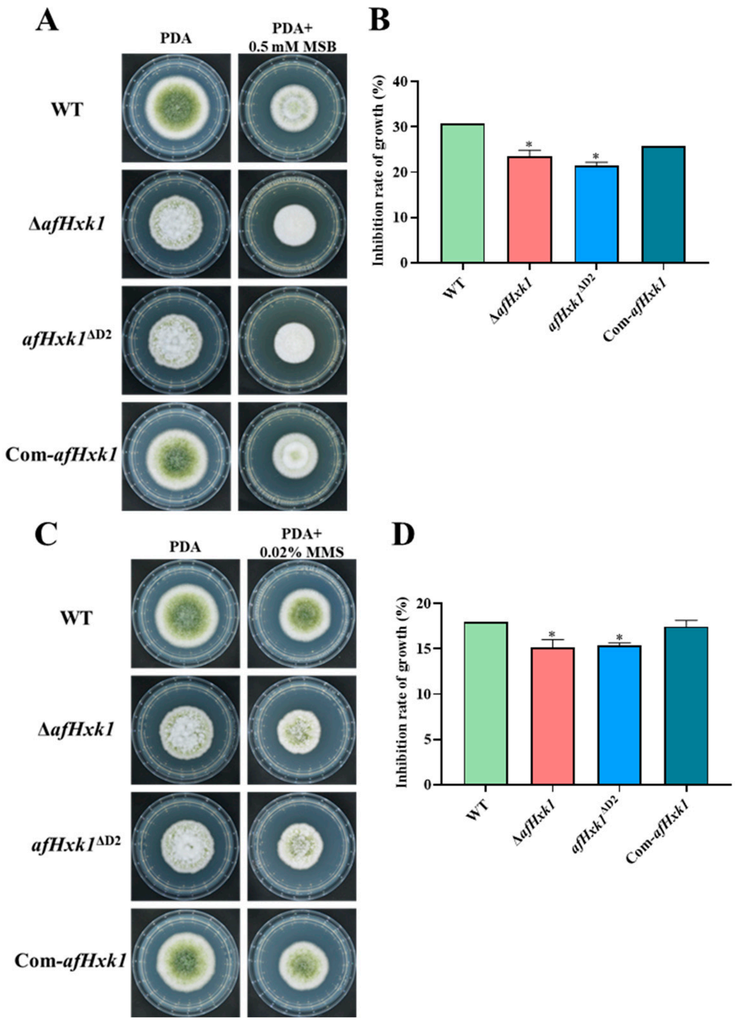

Figure S6. The impact of AfHxk1 on the response to oxidative stress and DNA damage in *A. flavus*. (A) WT, Δ*afHxk1*, *afHxk1*$^{\Delta D2}$ and Com-*afHxk1* strains were inoculated on PDA media with 0.5 mM MSB for 4 d. (B) The inhabitation rate of fungal growth under oxidative stress mediated by MSB. (C) The above fungal strains were inoculated on PDA with 0.02% MMS for 4 d. (D) The inhabitation rate of fungal growth under DNA damage stress mediated by MMS. * means significant difference $P < 0.05$.

Table S1. Strains used in this study.

Strain	Characterization	Source

A. flavus · CA14 PTS	Δ*ku70*, Δ*pyrG*	Purchased from FGSC
Wild type (WT)	Δ*ku70*, Δ*pyrG*:: *pyrG*	Prepared in our lab
Δ*afHxk1*	Δ*ku70*, Δ*Hxk1*:: *pyrG*	This study
afHxk1$^{\Delta D1}$	Δ*ku70*, Δ*Hxk1*:: *pyrG*, D1:: *pyrG*	This study
afHxk1$^{\Delta D2}$	Δ*ku70*, Δ*Hxk1*:: *pyrG*, D2:: *pyrG*	This study
Com-*afHxk1*	Δ*ku70*, Δ*Hxk1*:: *pyrG*, *Hxk1*:: *pyrG*	This study

Table S2. Primers used in this study.

Primer name	Sequence (5' to 3')	Fragment amplified
afHxk1-AF	GGGTGTTGAAACCTGAATGTAA	
afHxk1-AR	GGGTGAAGAGCATTGTTTGAGGCAAGCCGACATGGTCCGTTCA	
afHxk1-BF	GCATCAGTGCCTCCTCTCAGACCCACAGTCATCTATTTGTCGTT	Construction of Δ*afHxk1*
afHxk1-BR	ACGGGTTGCGTCTGCGGCGGAT	
afHxk1-NF	CCCGCAGATTGTTATCCGAGGT	
afHxk1-NR	AAGGATAGCAGGAAATCAAAGG	
Com-*afHxk1*-AF	ACGACCTACTTGAACGGACCA	
Com-*afHxk1*-AR	GGGTGAAGAGCATTGTTTGAGGCTCACGCCGATGTCTTTTC	
Com-*afHxk1*-BF	GCATCAGTGCCTCCTCTCAGACATTGCAGGGGAGATATAATCGC	Construction of Com-*afHxk1*
Com-*afHxk1*-BR	AAAGGAGTCTAGGCGGGAAG	
Com-*afHxk1*-NF	TGTTCCCCCTCTTGTAGTCG	
Com-*afHxk1*-NR	TGGAGAGGTGTTTGGTATGCT	
afHxk1$^{\Delta D1}$-AF	CTTTCCTGACGGTCTCCA	
afHxk1$^{\Delta D1}$-AR	GACATGCGGGTCTTGGGGTCAAGGTCATCGAGGGTACG	
afHxk1$^{\Delta D1}$-BF	TCGTACCCTCGATGACCTTGGACCCCAAGACCCGCAT	
afHxk1$^{\Delta D1}$-BR	GGGTGAAGAGCATTGTTTGAGGCTCACGCCGATGTCTTTTC	Construction of *afHxk1*$^{\Delta D1}$
afHxk1$^{\Delta D1}$-CF	GCATCAGTGCCTCCTCTCAGACCAGGGGAGATATAATCGCTT	
afHxk1$^{\Delta D1}$-CR	GTGTAATTGTTGCGTGAGGA	
afHxk1$^{\Delta D1}$-NF	AGGTCTCTCATTCACTATCGC	
afHxk1$^{\Delta D1}$-NR	GGACTGGCTGAAAGGAGTC	
afHxk1$^{\Delta D2}$-AF	AGGTCTCTCATTCACTATCGC	
afHxk1$^{\Delta D2}$-AR	TCTTTTCGGCCACAGCGGTCTTGGGGTCCACGTAA	
afHxk1$^{\Delta D2}$-BF	TTACGTGGACCCCAAGACCCGCTGTGGCCGAAAAGA	
afHxk1$^{\Delta D2}$-BR	GGGTGAAGAGCATTGTTTGAGGCCAAACAAGCCAGATGCCC	Construction of *afHxk1*$^{\Delta D2}$
afHxk1$^{\Delta D2}$-CF	GCATCAGTGCCTCCTCTCAGACGTTGTATCTTTGTAACACGGC	
afHxk1$^{\Delta D2}$-CR	GGAGTTAGAGCATTCAATTCG	
afHxk1$^{\Delta D2}$-NF	GAGAGTGCATAACTTCCGC	
afHxk1$^{\Delta D2}$-NR	GGCTGAAAGGAGTCTAGGC	
afHxk1-OF	AATCATTCCACAGGGCATC	To verify the existence of ORF
afHxk1-OR	CACGCCGATGTCTTTTCG	
P1020-F	ATCGGCAATACCGTCCAGAAGC	To verify the existence of AP and BP
P801-R	CAGGAGTTCTCGGGTTGTCG	
pyrG-F	GCCTCAAACAATGCTCTTCACCC	To amplify *pyrG*

pyrG-R	GTCTGAGAGGAGGCACTGATGC	
actin-qF	ACGGTGTCGTCACAAACTGG	To detect the transcription level of control gene
actin-qR	CGGTTGGACTTAGGGTTGATAG	
afHxk1-qF	ATGGACTGGGTGTCGCTT	To detect the expression level of *afHxk1*
afHxk1-qR	GCTATGCTATTGTTTCCATCAC	
abaA-qF	TCTTCGGTTGATGGATGATTTC	To detect the transcription level of conidia formation genes
abaA-qR	CCGTTGGGAGGCTGGGT	
brlA-qF	GCCTCCAGCGTCAACCTTC	
brlA-qR	TCTCTTCAAATGCTCTTGCCTC	
nsdD-qF	GGACTTGCGGGTCGTGCTA	To detect the transcription level of sclerotia formation genes
nsdD-qR	AGAACGCTGGGTCTGGTGC	
sclR-qF	CAATGAGCCTATGGGAGTGG	
sclR-qR	ATCTTCGCCCGAGTGGTT	
aflC-qF	GTGGTGGTTGCCAATGCG	To detect the transcription level of aflatoxin biosynthesis genes
aflC-qR	CTGAAACAGTAGGACGGGAGC	
aflD-qF	GTGGTGGTTGCCAATGCG	
aflD-qR	CTGAAACAGTAGGACGGGAGC	
aflO-qF	CTTTCGGCAGTGACCTAACC	
aflO-qR	TCTTGAACTATAAGGCGACCA	
aflR-qF	AAAGCACCCTGTCTTCCCTAAC	
aflR-qR	GAAGAGGTGGGTCAGTGTTTGTAG	
aflS-qF	CGAGTCGCTCAGGCGCTCAA	
aflS-qR	GCTCAGACTGACCGCCGCTC	
chsA-qF	TTTCCACATTCTCACGACAT	To detect the transcription level of genes related to cell wall stress mediated by CFW and CR
chsA-qR	TACCAATGTTGCGAGGTAAA	
chsB-qF	GTATCTCTCGGGAATGATCG	
chsB-qR	GAAGAAGTAGAGACCGACAC	
chsC-qF	AGCATCACCATGAAACTGAT	
chsC-qR	TCAGGACCTTGTCTTCATTG	
utr2-qF	GGTAAGGTAAAGAGTAGCCG	
utr2-qR	GGCAAACGAATCACTTACAG	
mnpA-qF	GACATCATCTCTGGCATCAG	
mnpA-qR	TGATATCATCACCGCTCTTG	
tcsB-qF	CATCTAAGGGTCAAGAGTCG	To detect the transcription level of genes related to osmotic stress mediated by KCl
tcsB-qR	CTTGACTACCGTTACATCGT	
skn7-qF	TCTCTGATCGTTATGCACAG	
skn7-qR	GTTGACGAGCATCTACTGAT	
sln1-qF	CATCTAAGGGTCAAGAGTCG	
sln1-qR	CTTGACTACCGTTACATCGT	
flbA-qF	TCCACGTTCACCAGCACTAC	To detect the transcription level of genes related to cell membrane stress mediated by SDS
flbA-qR	TACCTCCAATGGCCGAGAGA	
sfaD-qF	AGCTCGTCAAGGTTTGGGAG	
sfaD-qR	GGTGCTTGGACTCGTTGAGA	

Rho1-qF	TACCCCGACTCTCACGTCAT
Rho1-qR	TCTTGCGAACTTCCTCACCC
